# A Molecular Description of Hydrogel Forming Polymers for Cement-Based Printing Paste Applications

**DOI:** 10.3390/gels8090592

**Published:** 2022-09-16

**Authors:** Hajar Taheri-Afarani, Eugene Mamontov, William R. Carroll, Joseph J. Biernacki

**Affiliations:** 1 Department of Chemical Engineering, Tennessee Technological University, 1 William L. Jones Dr., Cookeville, TN 38505, USA; 2 Oak Ridge National Laboratory, 1 Bethel Valley Road, Oak Ridge, TN 37830, USA; 3 Department of Chemistry, Tennessee Technological University, 1 William L. Jones Dr., Cookeville, TN 38505, USA

**Keywords:** cement, Portland, 3D printing, additive manufacturing, neutron scattering, NMR, molecular dynamics, modeling, hydrogel, gel, admixture

## Abstract

This research endeavors to link the physical and chemical characteristics of select polymer hydrogels to differences in printability when used as printing aids in cement-based printing pastes. A variety of experimental probes including differential scanning calorimetry (DSC), NMR-diffusion ordered spectroscopy (DOSY), quasi-elastic neutron scattering (QENS) using neutron backscattering spectroscopy, and X-ray powder diffraction (XRD), along with molecular dynamic simulations, were used. Conjectures based on objective measures of printability and physical and chemical-molecular characteristics of the polymer gels are emerging that should help target printing aid selection and design, and mix formulation. Molecular simulations were shown to link higher hydrogen bond probability and larger radius of gyration to higher viscosity gels. Furthermore, the higher viscosity gels also produced higher elastic properties, as measured by neutron backscattering spectroscopy.

## 1. Introduction

Water-swollen polymeric networks, such as polymeric hydrogels, are complex dynamic systems [1]. Understanding how water interacts with gel-forming polymeric materials is critical to identify the underlying processes that make gels effective in specific applications [2,3]. A number of studies using molecular dynamic simulations [4,5] and experimental techniques, including nuclear magnetic resonance (NMR) spectroscopy [6,7,8], differential scanning calorimetry (DSC) [9,10,11], thermally stimulated depolarization currents (TSDC) [12], dielectric relaxation spectroscopy (DRS) [13], quasi-elastic neutron scattering (QENS) [4,14,15,16] and thermo-gravimetric analysis (TGA) [17], have been conducted to investigate the association and dynamics of water in polymeric hydrogels in various and unique applications. One such application is in construction materials [17,18,19,20]. The dynamics of the abovementioned techniques span a wide range of time scales. For instance, solvent and solute diffusion through network pores, segmental chain dynamics, matrix–solvent proton exchange, and local rotational state transitions differ by many orders of magnitude [1]. Despite significant progress in understanding the basic structure-property relationships of hydrogels, much remains to be learned about how the foundational macromolecular building blocks transmit properties across length-scales to the macroscopic sample. Fundamental grand challenges include understanding the relationship between network structure, dynamics, and mechanical properties [21]. One of the important factors is the state of water in such materials. Previous studies showed that water in hydrogels can generally be classified into three different categories [5,9,22,23]: freezable water (free water), non-freezable water (bound water), and freezable bound water. Free water behaves like bulk water. Free water freezes and thaws at 0 °C [5], though freezing may not be nucleated at 0 °C. Moreover, free water flows freely in hydrogels [5]. Although free water can be physically entrapped within the polymer network, it can be easily removed at ambient temperatures [24]. Free water does not participate in hydrogen bonds with the gel polymer molecules [24,25]. Freezable bound water is weakly bonded to the polymer chains or interacts weakly with non-freezable water. Freezable bound water undergoes a thermal phase transition at a temperature lower than 0 °C. Finally, non-freezable water is directly bound to the polymer chains through hydrophilic groups or via hydrogen bonds. Non-freezable water is strongly associated with polar groups of hydrogels by hydrogen bonds, which results in low mobility of non-freezable water [5]. As a result, such types of water cannot be easily separated from the hydrogel. During hydrogel swelling, water molecules that enter the polymer first form non-freezable water [5]. Additionally, non-freezable water does not exhibit a phase transition over the temperature range from −70 to 0 °C [5,9,22,23,24,25]. Different types of polymers show their own unique characteristics when in contact with water molecules. These characteristics lead to various effects on hydrogen-bonding, due to the presence of different types and contents of water molecules based on the aforementioned categories. Therefore, determining the content of water in the abovementioned categories is crucial for understanding the properties of hydrogels and their dynamic behavior. Furthermore, the dynamics of water is highly affected by the confinement environment, e.g., mesoporous silicas (MCM-41) [26], carbon nanotubes, mesoporous organo-silicas, zeolites [27] and cementitious systems [28,29].

There has been widespread interest in the structure and dynamics of water in confined media and the influence of confinement on hydrogen bonding, interfacial transport, and mechanical toughness of the surfaces [30]. Therefore, studying the association of water confined in hydrogels might provide useful information on the molecular structure, interfacial properties, interactions, and the dynamic behavior of such materials [4,31,32,33]. However, there are still few studies based on polymeric hydrogels confined within hard walls, such as in the abovementioned structures. Selected hydrogel-forming polymers have recently been used to produce cement-based printing pastes with good printability outcomes [34,35]. Yet, the interaction of these hydrogel-forming polymers with cement and other types of hard walls and the dynamic behavior of water in such systems have not been investigated thoroughly. Thus, a comprehensive description of such systems requires a range of experimental approaches and interpretive models. For instance, QENS [36] was used to study the dynamics of both water and poly N-isopropylacrylamide (PNIPA) molecules in concentrated PNIPA solutions. In that system it was shown that hydration plays an important role for the stabilization of the aqueous system [36]. Results showed that when water and cement materials interacted with each other, the commencement of the hydration reaction allows free water to convert to chemically bound water which is representative of the motionless state of water. For systems including polymeric hydrogels, the formation of hydrogen bonding between polymers and hydrating water molecules reduces the free energy of the solutions [36]. QENS was also used to explore early-age hydration kinetics of Portland cement with nano-additives, such as nano-silica (NS) [37].

Small-angle neutron scattering (SANS) is another promising approach to elucidate properties of hydrogels and their network structures [21,38]. SANS studies have been used to characterize the shape, size, and dimensions of gel fibers [39,40,41], e.g., SANS was used to investigate the microstructure and relative homogeneity of tetra-functional poly ethylene glycol (PEG) networks [21]. Qualitative analysis and model-fitting of SANS data showed that at the concentrations of 35 kg.mol^−1^ and 12 kg.mol^−1^ tetra-functional PEG hydrogels have a remarkably homogeneous network structure with low junction functionality [21]. In another study, the rheological and structural properties of a pharmaceutical multicomponent hydrogel, consisting of a hydrogel-forming (acrylic acid) polymer (Carbopol^®^ 974P, BF Goodrich Europe, UK), microbicide (monocaprin, Danisko Ingedients, Denmark), nonionic surfactant (Tween 20^®^ or Tween 40^®^, Sigma Chemical Co., St. Louis, MI, USA), and preservatives, was characterized using rheological measurement and SANS [42]. The rheological measurements showed that the formation of large-scale structures was weakened at higher pH values. These results agreed with the SANS results which showed that at high system pH, repulsive electrostatic forces reduce the tendency to form chain associations, whereas at low pH the electrostatic repulsive forces are suppressed in the hydrogel, and this leads to the association and the production of large-scale structural heterogeneities [42].

QENS measurements using neutron backscattering spectroscopy (such as those performed at BASIS [43]), is also a capable approach for measuring a dynamic spectrum near the elastic peak of around ± 100 µeV in the energy transfer with an elastic energy resolution of 3.7 µeV (full width at half-maximum value averaged over all scattering angles) [44]. Due to its large dynamic range and high energy resolution, BASIS is a suitable instrument for studying dynamics of confined supercooled water.

Molecular dynamics simulation (MDS) techniques have also been used to characterize the molecular structure and properties of various materials [5,45,46,47]. Such studies can provide detailed information about the structure and behavior of systems on the molecular level. To this end, a molecular dynamic simulation was carried out [5] to explore the content of non-freezable water, freezable water, and free water at various degrees of cross-linking for poly (vinyl) alcohol (PVA) [5]. The simulation results showed that the amounts of non-freezable water and freezable water were slightly less than those found experimentally [5]. To better understand the interaction between water and polymers and the role of hydrogen bonds in hydrogels, MDS of PVA, poly (vinyl methyl ether) (PVME), and PNIPA was used [45]. Results suggest that the largest numbers of hydrogen bonds occurred between water and polymers for PVA (the more hydrophilic polymer), while the smallest numbers were observed for PVME (the more hydrophobic polymer) [45]. Since PVA is more hydrophilic than PVME and PNIPA, the greatest numbers of water molecules were found to be non-freezable. However, for PVME, the most hydrophobic polymer, a larger amount of water was free water [45].

One of the most interesting features of hydrogels is their tribological properties. Tribology refers to applying the concepts of friction, lubrication and wearing to study interacting surfaces in relative motion [48]. Hydrogels have been used as anti-friction materials in various industries [49]. Methyl cellulose and its derivatives, for example, are hydrogels that have been used to provide lubrication properties which are important in the cement extrusion industries [50,51,52,53,54]. To better understand the tribological properties from an atomistic point of view, MDS was used [55] to examine the role of sliding orientation on the tribological properties of polyethylene (PE). PE surfaces were slid against each other in two different directions: (1) perpendicular to the aligned direction of the polymer chains and (2) parallel to the chains. Sliding in the parallel direction showed a lower friction coefficient than sliding in the perpendicular direction [55]. Greater levels of shear strain were also stored during sliding in the perpendicular direction. Furthermore, their results suggested that the friction coefficient was dependent on the local orientation of polymer chains with respect to the sliding direction and molecular motion.

In the authors’ previous works, rheological properties, including flow properties of hydrogels made from hydroxy ethyl methyl cellulose (HEMC), hydroxy propyl methyl cellulose (HPMC) and poly ethylene oxide (PEO), and oscillatory rheology of the neat gels and gel-cement pastes, were correlated to the printability of the same paste systems using rigorous techniques for quantification of print fidelity [34,35]. A number of inferences emerged: (1) printing outcomes in order of best to worst were for gel-based pastes made with HEMC, HPMC and PEO, (2) neat gels exhibited shear time-independent shear thinning behavior, (3) There was an inverse relationship between the storage modulus of the neat gel and the storage modulus of the gel-based cement pastes, i.e., higher gel storage modulus produces lower paste modulus and (4) pastes with higher gel modulus were preferred and produced better printing outcomes at the same polymer contents. Notably, the range of polymers that produce hydrogels is broad; however, not all hydrogels are applicable as cement-paste printing aides. The three gels used in the prior work of the authors exhibited both mechanical and chemical stability when used in aqueous suspension of Portland cement. Such gels must remain stable under conditions of high shear, which are imposed during mixing and must be stable in the high pH environment of Portland cement, i.e., pH of up to nominally 12.5. Hydroxylated celluloses and PEO are representative polymers with different chemical architectures and so were selected as case study materials. In the present study, HEMC, HPMC and PEO were again used to form gels, thus, extending the prior work of the authors [34,35]. In order to elucidate how water interacts with polymeric hydrogels, as well as with other materials, such as cement, MDS were conducted alongside experimental probes. This study intends to look at molecular scale connections between gel structure and printability. Thus, in the current work, the BASIS spectrometer at Oak Ridge National Laboratory (ORNL) was used to compare the structure of hydrogels, the phase behavior, and the dynamics of water in neat cement pastes and water in cement pastes made using hydrogels. Moreover, diffusion-ordered nuclear magnetic resonance (DOSY-NMR) spectroscopy was also used to study the mobility of polymers and water in the gel phase and differential scanning calorimetry (DSC)-based thermoporometric measurements were made to investigate the porous structures of the hydrogels. By studying water dynamics in cement paste and in hydrogels at the molecular level, basic information is provided for the formulation of new additive platforms and to engineer additives for specific applications in the cement industry.

The following discourse first looks at the characterization of the three polymer types and the gels formed therefrom, systematically discussing the findings by the following applied test methods: X-ray diffraction, differential scanning calorimetry, neutron scattering and, finally, molecular dynamic simulation. The results are followed by a description of the experimental methods and computational procedures. The present study is concerned only with the selection of gel-forming polymers for printability and does not address the issues of properties of the printed composites, e.g., strength or durability. Print quality, however, is addressed in the authors prior studies wherein the gel-cement pastes were printed and the printed objects were characterized for print fidelity [34,35], i.e., how the dimensions of the printed objects compared to that of the model or target object.

## 2. Results and Discussion

### 2.1. Microstructural Analysis

**Figure 1:**
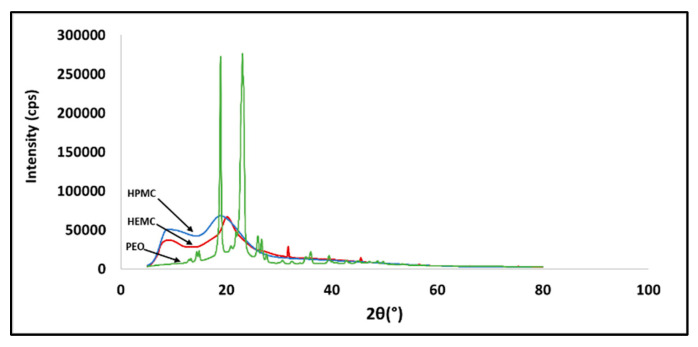
X-ray diffraction pattern of HEMC, HPMC, and PEO.

X-ray powder diffraction (XRD) patterns of the three raw polymers are shown in Figure 1. HEMC was found to have a semi-crystalline structure, HPMC was totally amorphous, and PEO was completely crystalline. The XRD pattern of PEO showed two high intensity diffraction peaks at 19.36° and 23.72°, which were assigned to the (120) and (112) planes, respectively [56]. While this information was interesting, it is provided here for information only and as a form of characterization of the raw material, since, once infused with water, all three gel types were amorphous.

### 2.2. Differential Scanning Calorimetry (DSC)

**Figure 2:**
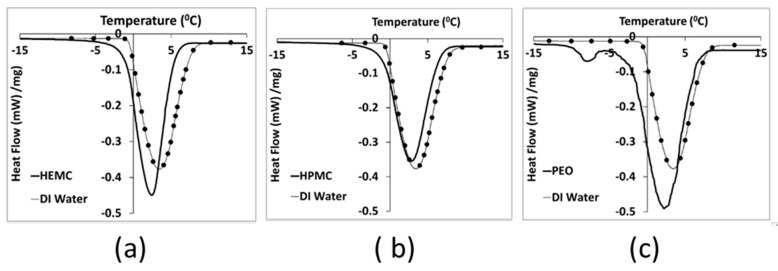
DSC heating curves for: (**a**) Gel 1 (HEMC), (**b**) Gel 2 (HPMC) and (**c**) Gel 3 (PEO), compared to deionized water, i.e., line with dots.

Differential scanning calorimetry-based thermoporosimetry of the three hydrogels at 4 mass % polymer content was used to study the qualitative pore structure and association of water and polymer in the gels. The melting curves of all three hydrogels displayed a broad primary endothermic peak [8,57], shown in Figure 2. PEO hydrogels also exhibited a small peak at about −8 °C. This small peak might be assigned to the melting of a small amount of PEO trihydrate complex that crystallized during the cooling step [57,58,59]. Therefore, it might be considered as non-freezing water, i.e., water that was strongly bonded to the polymeric chains. Melting of pure water starts at 0 °C ± ½ °C, while for the three hydrogels melting was initiated at between −10 °C and −7 °C [60]. Moreover, since melting began below the normal temperature of fusion for water, this indicated a porous structure, i.e., water confined within small gel pores. Confined water has a depressed freezing point [61], and the finer the pore structure, i.e., the smaller the pores, the greater the freezing point depression, as indicated by the Laplace equation [62,63].

PEO appeared to have the finest pore structure, while HPMC had the coarsest structure, as seen by comparing the thawing curves for the gels to that of DI water. HEMC exhibited an intermediate pore structure, while it also exhibited better printability results in comparison to that of HPMC and PEO. Therefore, gel pore structure alone does not appear to be a direct indicator of paste printability, i.e., printability does not monotonically increase or decrease with changes in pore fineness as measured by freezing point depression. Gel pore structure, however, is one indicator of gel-to-gel differences that can be considered alongside other metrics in the search for connections between molecular structure and printability.

### 2.3. NMR DOSY Experiments

**Figure 3:**
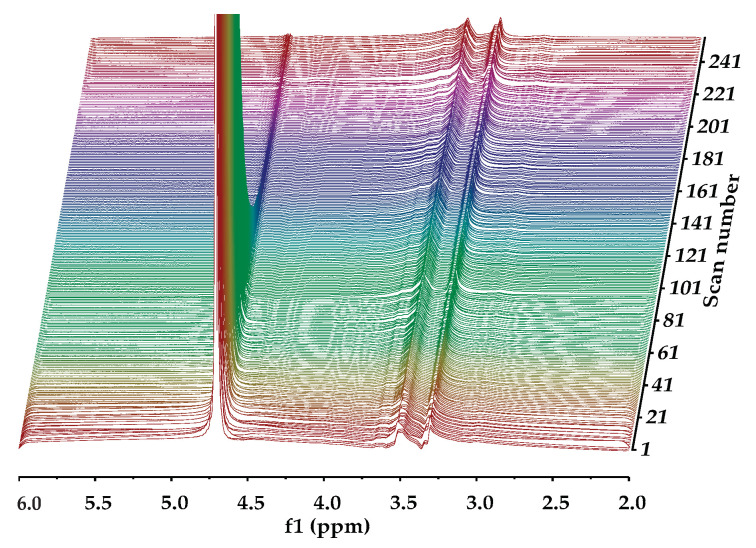
NMR DOSY experiment for the 0.5 mass % HEMC hydrogels: a series of NMR diffusion spectra with varying gradient strengths. Chemical shifts (in ppm) lie on the horizontal axis while magnetic gradient pulse amplitude is on the orthogonal axis, increasing with increasing scan number.

Diffusion ordered NMR spectroscopy was used to investigate the self-diffusivity of polymer and residual water in the gels [64,65]. DOSY experiments for 0.5 mass % HEMC, 2.0 mass % PEO, and 0.2 mass % PEO gels were carried out, since the polymer structures were different. Figure 3 presents the signal intensity decays of the deuterium oxide (HOD solvent residual in D_2_O) and polymer molecules present in the hydrogels, in relation to the increasing field gradient strength as the scan number increased. Such signal attenuation is dependent on several parameters, including the original signal intensity (concentration), the gradient pulse area, the diffusion time, and the diffusion coefficient of the molecule [66,67]. It is clear that NMR signals, due to different diffusing species, are attenuated at different rates. As mentioned before, this is due to different diffusion coefficients, i.e., the signals at 3.5 and 4.8 ppm belong to HOD and polymer molecules which are diffusing at different rates. The decay of the signal originating from the residual solvent (HOD, δ 4.8 ppm) was fast, again refer to Figure 3, which is an indicator of mobility.

Whereas the signal from the solvated polymer (multiple peaks around δ 3.5 ppm) did not decay during the timeframe of the experiment. This indicated that the polymer molecule moved slowly relative to the free HOD. The diffusivity of the water molecules (HOD) was found to be 2.61 × 10^−9^ m^2^/s in HEMC gels, respectively. Notably, the self-diffusivity of free water at the same temperature is 2.299 × 10^−9^ m^2^/s [68]. The diffusivity of solvated dissolved HEMC was found to be 3.13 × 10^−11^ m^2^/s. The solvated HEMC appearing in the solution state NMR data presented here represented molecules much larger than water. It was consistent with our expectation that it would diffuse more slowly than water. It is expected that any solid HEMC would experience line broadening, due to dipolar couplings, that prevent it from providing a clear solution state NMR signal.

**Figure 4:**
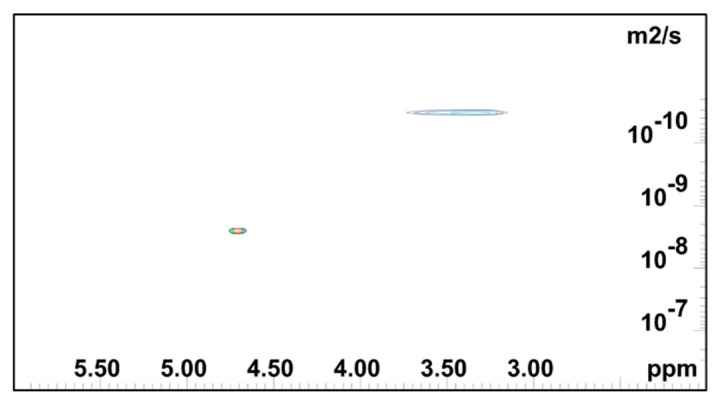
2D DOSY log(D) plot for HEMC 2 mass % gel.

**Figure 5:**
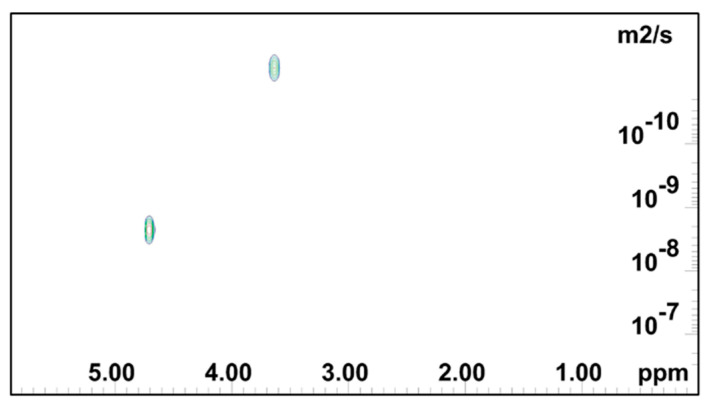
NMR DOSY log(D) plot for 0.2 mass % PEO gel.

**Figure 6:**
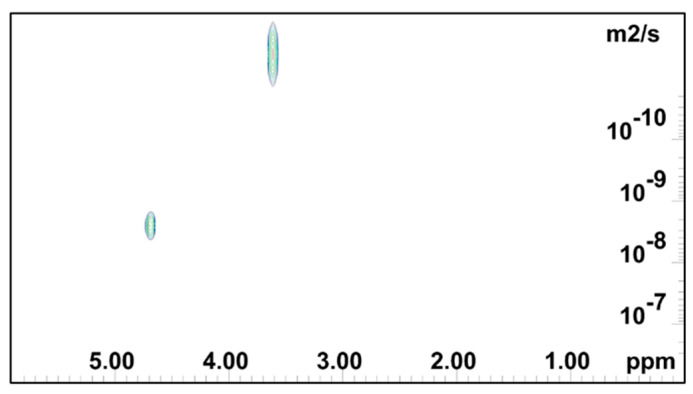
2D DOSY log(D) plot for PEO 2 mass % gel.

Figure 4, Figure 5 and Figure 6 show the 2D DOSY NMR spectra of 0.5 mass % HEMC, 0.2 mass % PEO, and 2.0 mass % PEO solvated with D_2_O. ^1^H NMR spectrum and the diffusion coefficient domain are represented on the x- and y-axes, respectively. In each plot there was an observable solvent residual peak around δ 4.8 ppm and a signal for free solvated polymer, either HEMC or PEO, at δ 3.5 ppm and δ 3.6 ppm, respectively. Both high concentration PEO samples (2.0 mass %) that formed a gel and lower concentration PEO (0.2 mass %) samples forming a vicus solution were examined. In each case, as in the HEMC case, water diffused at nominally the bulk water self-diffusivity, with 0.2 mass % PEO samples showing 2.32×10^−9^ m^2^/s and 2.0 mass % PEO samples showing 2.45 × 10^−9^ m^2^/s. Notably, the line-broadness was not the same for each. PEO samples had a broader signal for HOD and for the polymer, observed in the diffusion domain. This was consistent with DSC thermoporosimetry outcomes that clearly indicated a confined water environment in all three gel types with greater confinement in the PEO gels. Water participating in several chemical environments displays a diversity of diffusion coefficients and chemical shifts not seen in bulk water. Figure 6 shows the DOSY PEO results for 2.0 mass % solution in D_2_O. These results were also consistent with DSC findings that suggested that PEO has a finer pore structure than HEMC. As mentioned above, however, this finer pore structure alone does not necessarily translate to good or bad printability outcomes; however, the data elucidates quantifiable differences in pore structure. Observable in this spectrum were further broadened signals beyond those observed in the lower concentration sample. Similarly to HEMC, the polymer signals in the PEO samples diffused more slowly than those of HOD.

### 2.4. Neutron Scattering

#### 2.4.1. Energy-Resolved “Elastic” Scattering

**Figure 7:**
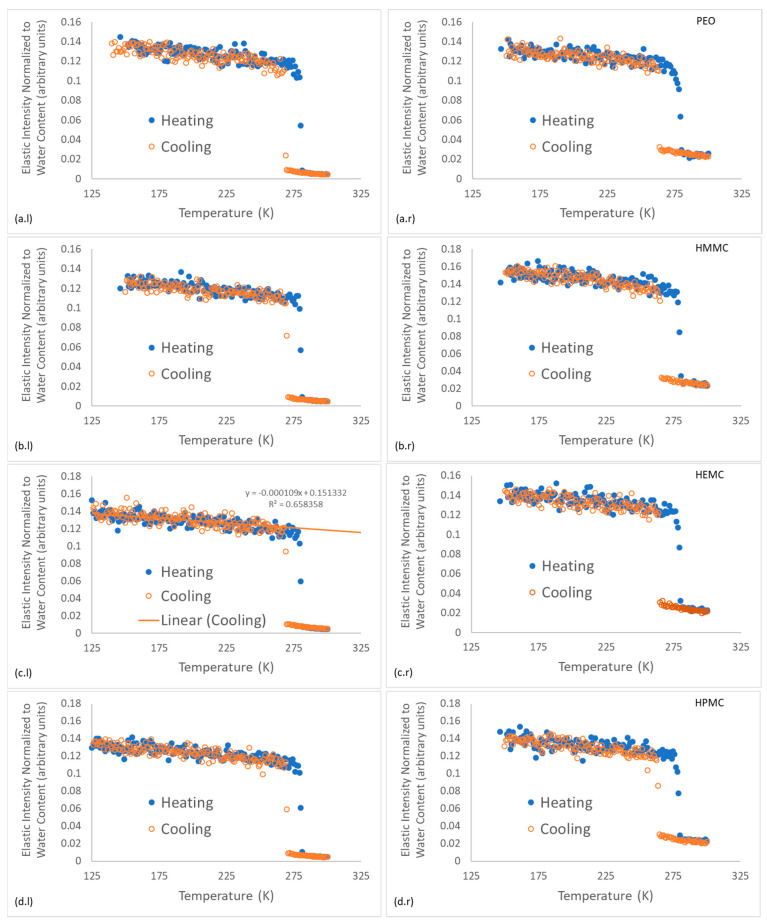
Energy-resolved “elastic” scattering data for hydrogels and hydrogel-cement pastes; the cooling curves are plotted with solid markers and the heating curves are plotted with open markers. Figures on the left (**l**) are the neat gels and figures on the right (**r**) are the gel-cement pastes. Subfigures are shown for each gel type: (**a**) PEO, (**b**) HMMC, (**c**) HEMC and (**d**) HPMC.

**Figure 8:**
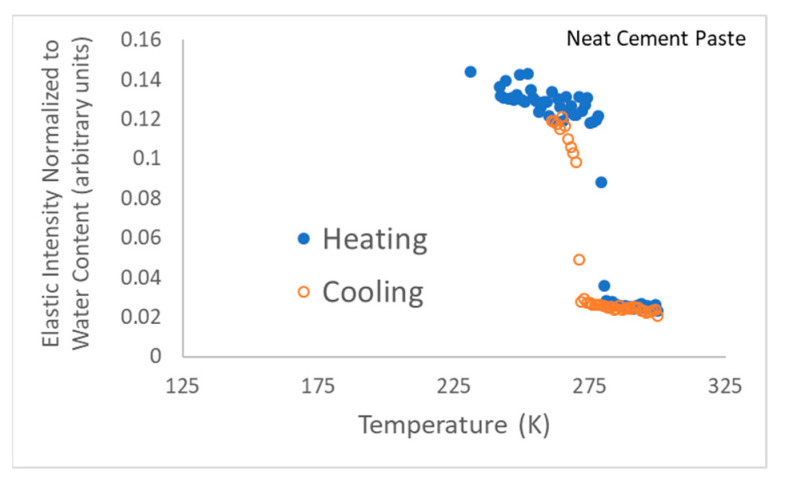
Energy-resolved “elastic” scattering data for neat cement paste, i.e., without gel. Note that this neutron scattering run was terminated just after the phase transition since the team’s neutron beamtime was running out.

The “elastic” scattering intensity (due to neutrons scattered from the sample with an energy transfer within the FWHM of the energy resolution line) was recorded on both cooling of samples and on heating. Refer to Figure 7 for gel and gel-cement paste data and to Figure 8 for neat cement paste data. The raw elastic intensity was normalized with respect to water content, i.e., mass of water. For the pure gel samples the raw intensities were divided by 0.38 g. For the gel-cement samples, a normalization factor of 0.18 g, i.e., 20% of the 4:1 cement: gel sample mass, was used.

The energy-resolved “elastic” intensity scans provide insights into the gel-water structure and dynamics. As expected, a phase transition for the freezing of water was observed for all samples between 263–271 K and was characterized as an abrupt change in elastic intensity as the scattering centers became effectively immobile. Further cooling resulted in a continuous and monotonic increase in elastic intensity with decreasing temperature for all materials, as shown for HEMC in Figure 7c.l. Upon heating all samples demonstrated melting, indicated by an abrupt decrease in elastic intensity at about 275 K, i.e., near the melting point of water, 273.15 K.

Figure 7 illustrates that some supercooling before freezing was evident in all the gels, though a larger supercooling was required for the gel-cement samples, with only small variations. Gel-cement samples freeze at a temperature between 263 K and 265 K, while neat gels freeze between 268 K and 271 K. PEO froze at the notably lowest temperature of 268 K, a finding that was both consistent with the DSC results, indicating a finer pore structure, and the NMR DOSY results, which indicate the same. Very little variation was observed for melting.

**Figure 9:**
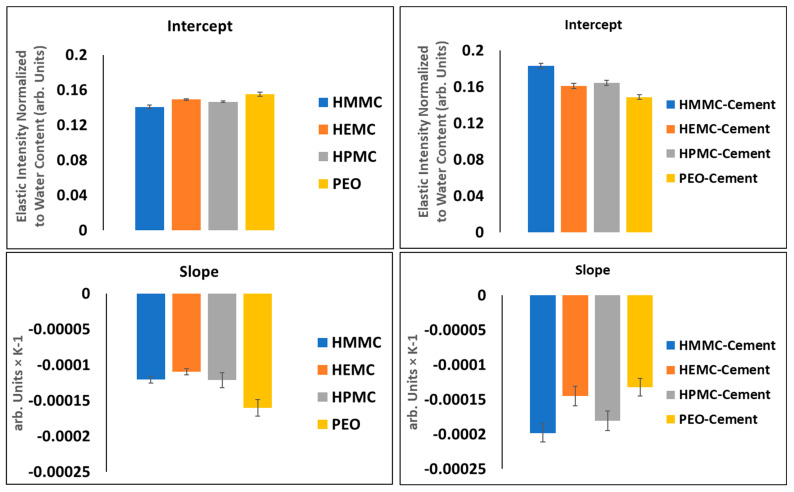
Regression analysis of linear region of cooling section for gel (**left-side**) and gel-cement pastes (**right-side**).

For clarity, the “elastic” intensity data for each hydrogel was plotted separately; all the individual figures are shown, refer to Figure 7 which presents the plots of normalized elastic intensity as a function of temperature for the entire 4 mass % gel series, including PEO, HMMC, HEMC and HPMC. The linear region of the cooling section was fit with a straight line, as shown on Figure 7c.l as an example. The slope and intercept and respective standard errors were obtained. The resulting values are compared in the form of bar charts, refer to Figure 9. This data provides information about the structural characteristics of the gel and pastes, albeit in the frozen state. The intercept is a measure of the amount of “elastic” scattering, i.e., the intensity of “elastically” scattered neutrons. The higher the intensity, the greater the number of “elastic” scattering sites. In this case, the majority of the elastic scattering came from the frozen water. For gels, the amount of frozen water was nominally constant, gel-to-gel, since the water content of the gels was constant at 4 mass %. Furthermore, the elastic intensity had been normalized to water content. Thus, the intercept data, which for the neat gels indicated little or no variation with polymer type, said little about the effect of the polymer on the number of elastic scattering sites.

The slope, however, is the rate of change of intensity with respect to temperature and is proportional to the Debye-Waller (DW) factor which is a function of a material’s elastic modulus, “*a*,” i.e., the elastic constant of the vibrating “spring” that binds each atom to its equilibrium position, refer to Equations (1)–(3) [69]. For the linear portion of the *I(T)* vs. *T* curve, the quotient of the elastically scattered intensity at some *T* and some reference *T_0_*, is defined as the Debye-Waller factor, refer to Equation (1). By transforming Equation (1) using the definition of the linear slope of the *I(T)* vs. *T* line, the Debye-Waller factor thus becomes a function of the slope, hence relating the material modulus to slope, refer to Equation (3). It is easily seen that for a perfectly rigid material, the slope must be zero. The relative stiffness of gels and gels confined within the gel-cement pastes are indicated by comparing the slopes of the *I(T)* lines; stiffer materials have lower slopes. The data indicated that the neat HEMC was stiffer than other hydrogels. This finding was consistent with rheological data which showed that HEMC had higher viscosity than either HPMC or PEO [34,35].

(1) $$\frac{I\left(T\right)}{I\left(T_{0}\right)}=DW=exp\left\{-\frac{12\pi^{2}k}{ad^{2}}\left(T-T_{0}\right)\right\}$$

(2) $$s=slope=\frac{I\left(T\right)-I\left(T_{0}\right)}{T-T_{0}}$$

(3) $$s\frac{\left(T-T_{0}\right)}{I\left(T_{0}\right)}+1=exp\left\{-\frac{12\pi^{2}k}{ad^{2}}\left(T-T_{0}\right)\right\}$$

where, *k* is the Boltzmann constant, *d* is the atomic spacing, *T* is absolute temperature and the subscript “*0* ” indicates some arbitrary reference state [69].

#### 2.4.2. Dynamic Scattering

QENS dynamic scattering data measured at BASIS is in the form of the scattering function, I (Q, E), and this can be understood as the convolution of the dynamic structure factor with the instrumental resolution function, refer to Equation (4) [70]. In the incoherent approximation, this function is related to the self-correlation function of the water protons in time and space through the Fourier transform from frequency (energy) to time and from wave-vector (Q) space to real space [70], as shown here:

(4) I(Q,E)=[x·δ(E)+(1−x)·S(Q,E)]⊗R(Q,E)+B·E+C

where, *Q* (the momentum transfer) can be obtained [71] from:

(5) hQ=h(ki−kf), ki=2πλi,kf=2πλf

and where *I (Q, E)* is the neutron scattering intensity as a function of momentum transfer and energy, *h* = 4.135 × 10^−15^ eVs is Planck’s constant, *λ* is the neutron wavelength, *x* is the weighting fraction of the elastic component approximated by the function *δ(E)*, *S(Q, E)* is the Lorentzian dynamic structure factor corresponding to the sample (i.e., the Lorentzian function), *R(Q, E)* is the resolution function of the spectrometer and ⦻ is the numerical convolution [72]. The BASIS raw dynamic scattering data were fit with a resolution-convolved Lorentz function plus a linear background term of (*B·E + C*) for the gel samples and a superposition of a resolution-convolved Lorentz function and a delta-function (*δ* (*E*)) for elastic, or “settled water” intensity plus a linear background for the cement and gel-cement samples. The experimental *I(Q, E)* was fit using a single Lorentzian function *S(Q, E)* where *Γ(Q)* is the half-width at half-maximum (HWHM) [72,73,74]:

(6) $$S\left(Q,E\right)=\frac{1}{\pi }\cdot \frac{\Gamma \left(Q\right)}{E^{2}+\Gamma^{2}\left(Q\right)}$$

**Figure 10:**
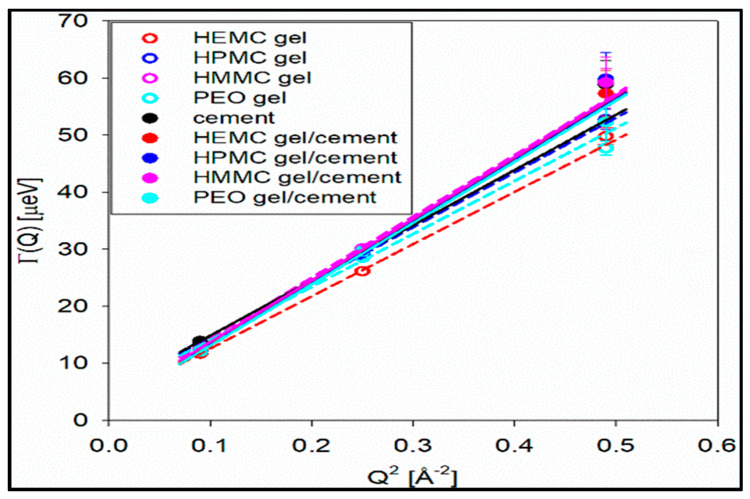
Plot of Lorentzian HWHM versus *Q*^2^ for all samples.

The half-width at half-maxima (HWHM) of the Lorentzian function is proportional to *Q*^2^ and, thus, provides a well-established method for determination of diffusion coefficients [72,73,74]. The dynamic range of BASIS (3.7 to 200 μeV) is suitable for studies of water diffusion [72]. The resulting half-width at half-maxima (HWHM(Q^2^)) of the Lorentzian functions are presented in Figure 10. From these data, the water diffusivities, *D*, were obtained from a linear fit of HWHM(Q^2^) with a (*Q*^2^ + offset) function using Equation (7) [72,73,74]. The non-zero offset term was due to multiple scattering effects in the samples.

(7) $$D_{s}\left(Q\right)=\frac{\Gamma \left(Q\right)}{hQ^{2}}$$

**Figure 11:**
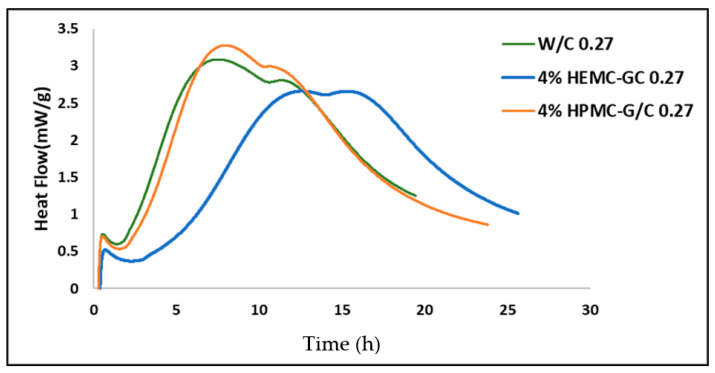
Hydration Calorimetry results for neat cement and gel-cement pastes containing 4 mass % HEMC and HEMC at a gel/cement ratio of 0.27.

Table 1 shows the diffusivities and the “elastic”, or “settled”, water fractions for the cement and gel-cement samples. Hydroxy methyl methyl cellulose (HMMC) was included in the series, expanding the study to four gels, to provide a more complete description of how properties change with the cellulose derivative moiety. The half-width at half-maxima of the *S(Q, E)* spectra obtained from the model fit [72,73,74] shows a strong *Q* -dependence, refer to Figure 10, confirming a long-range translational diffusion process for water molecules in the samples. It was evident that there was an increase in the *Γ* for the gel-cement pastes compared to that of the neat gels. From Table 1, it is clear that the diffusion coefficient of water molecules in HMMC gel was greater than for the other three gels. However, the water diffusivity was almost the same for pastes, and independent of gel type. It was notable that the water diffusivity in the cement paste was lower than in gel-cement-based hydrogel pastes. This might be attributed to the hydration effect. As is shown in Figure 11, the gel-forming polymers used in this study had a complex effect on hydration. It was notable that HEMC both slowed the rate of hydration and delayed the onset of rapid hydration. For the neat cement paste, the rate of diffusivity of water was slightly lower, which might have been due to the higher extent of hydration of the neat cement, again refer to Figure 11. From Table 1, the elastic signal fraction of neat cement was somewhat higher than cement-based hydrogel pastes, which might be evidence that neat cement was more hydrated and, thus, the observed water diffusivity was lower.

The diffusivity in the gel-cement samples was somewhat higher, on average, than the diffusivity in the corresponding gel samples. Compared to the neat cement sample, the gel-cement diffusivities were also somewhat higher, and the elastic (settled) water fractions somewhat lower. It should be noted that the pure bulk water diffusivity value at 300 K was ca. 24 × 10^−10^ m^2^/s [72].

### 2.5. Atomistic Simulations

**Figure 12:**
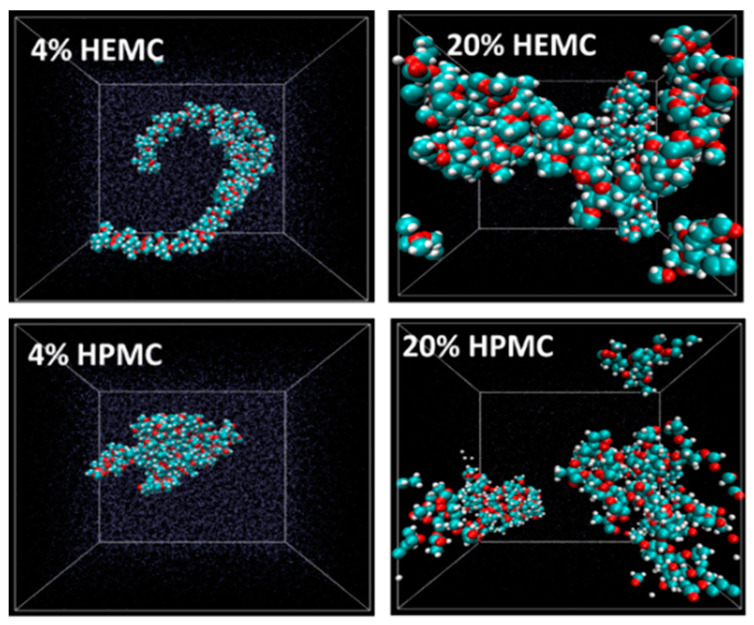
Conformation of HEMC and HPMC at 4 mass % and 20 mass %; O-red, C-blue and H-white. Box size is square and constant at 4.2 nm (42 Å).

In support of the experimental probes, molecular-scale simulations were also done. Atomistic simulations were performed on the cellulose derivative hydrogels only, i.e., HEMC and HPMC, in order to study their molecular behavior. The initial monomer structures were built using Materials Studio (Accelrys, now BIOVIA) while the data runs were made with LAMMPS (Large-scale Atomic/Molecular Massively Parallel Simulator, a free and open-source software developed by researchers at Sandia National Laboratory (SNL) and Temple University). H_2_O molecules were added using the Grand Canonical Monte Carlo (GCMC) technique [75,76,77]. Each system was equilibrated using an NVT (constant number, volume and temperature) canonical ensemble at 298.15 K. The systems were relaxed using an NPT (constant number, pressure and temperature) ensemble, and, finally, the systems were again equilibrated using an NVT ensemble. These MDSs focused on polymer conformation, mobility of both water and polymer and hydrogen bonding structures. Polymer conformations were investigated at different concentrations. The equilibrated molecular structures for 4 mass % and 20 mass % HEMC and HPMC are shown in Figure 12 with water rendered as points. Inferences from images like these suggest that HEMC formed a more swollen, open, or dispersed structure than did HPMC. This more open structure might be partly responsible for HEMC’s better performance as a printing aid. It is also likely responsible for the higher viscosity as well.

**Figure 13:**
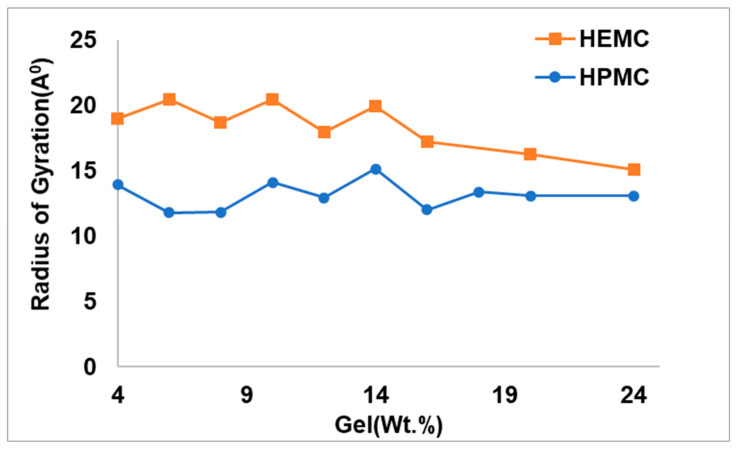
Radius of gyration of HEMC and HPMC at different polymer concentrations.

Radii of gyration (Rg), a more direct indicator of the extent of swelling or openness of the molecule, were calculated for different polymer concentrations. The radius of gyration of a dissolved polymer provides an indication of the polymer’s size in that environment. Figure 13 shows the Rg of the gel-forming polymers as a function of polymer concentration for HEMC and HPMC. For all concentrations, the size of the HEMC polymer was greater than the HPMC polymer. With a periodic simulation box of size approximately 42 Å, the Rg for HEMC indicated that the polymers spanned the entire box forming a percolated network.

**Figure 14:**
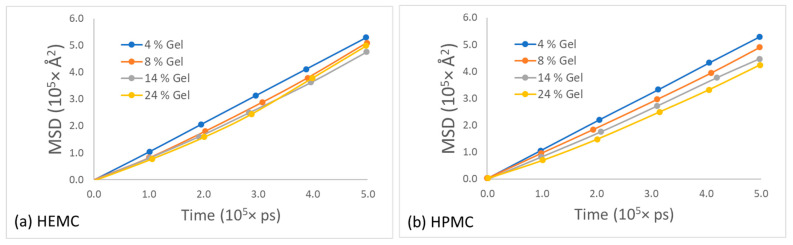
Mean square displacement of water for different polymer concentration gels for: (**a**) HEMC and (**b**) HPMC.

To obtain the self-diffusivity of water at different concentrations, the mean squared displacement of the center of mass of water molecules in HEMC and HPMC gels was plotted as a function of time and parametrically as a function of gel polymer mass percentage, refer to Figure 14. For both polymers, the mobility of water decreased with an increase in polymer concentration, as expected. However, the drop in the mobility of water in HEMC was larger than that in HPMC. This was consistent with the gel rheology experiments that showed a higher viscosity for HEMC.

**Figure 15:**
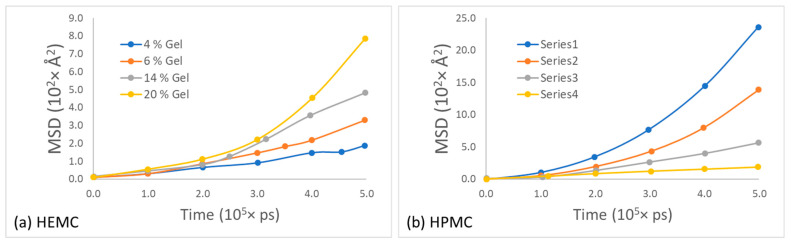
Mean square displacement of polymer at different concentrations of gel for: (**a**) HEMC and (**b**) HPMC.

Likewise, the mean squared displacement of polymer centers of mass for the two gels at various concentrations is shown in Figure 15. The most apparent observation from this data was that the mobility of these two polymers appeared similar at low polymer content, while the HPMC mobility appeared greater than that of HEMC at high polymer content. One explanation for this was consistent with the qualitative observation that HEMC formed a more percolated network than HPMC, and, thus, HEMC molecules had more restricted motion than HPMC, particularly at higher concentrations. This was also consistent with the experimentally measured viscosities.

**Figure 16:**
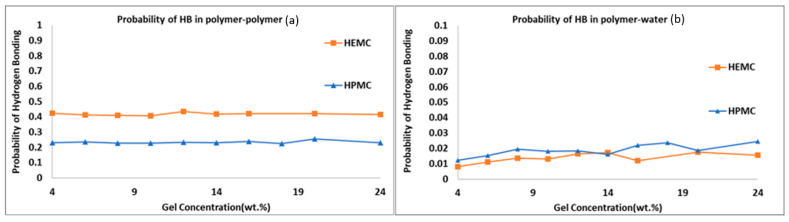
Probability of hydrogen bonding: (**a**) polymer-to-polymer, and (**b**) polymer-to-water.

To investigate the effect of hydrogen bonding on printability results, the probabilities of hydrogen bonding within the polymer and between polymer and water were calculated, refer to Figure 16. Here, the probability was defined as the number of hydrogen bonds actually formed divided by the total number of hydrogen bonds possible in a given system. The probability of hydrogen bond formation within the polymer was highest for HEMC, refer to Figure 16a. However, the trend was reversed for hydrogen bonds between polymer and water, with more hydrogen bonds between polymer and water observed for HPMC than HEMC, though marginally higher, refer to Figure 16b. These finding were again consistent with the observed viscosity of the gels and indicated that the greater number of hydrogen bonds within the polymer resulted in lower mobility of the chains and higher viscosity, as in this case for HEMC.

## 3. Conclusions

The gels of three hydrogel-forming polymers, which were previously used as printing aids for cement-based pastes, were characterized using a range of experimental and computational techniques that bridged a wide domain of length scales. Prior experimental studies showed that polymers that form more viscous gels produce better printing outcomes. Furthermore, those same gels produce cement-based printing pastes with lower storage moduli. The present multi-scale analysis showed that higher gel viscosity was produced by polymers that were more swollen, i.e., having expanded, polymeric networks, and which correlated to larger radii of gyration. These hydrogels seem to form more intramolecular hydrogen bonds, i.e., more entangled structures, and are more favorable as printing aids, since the high degree of polymer-to-polymer bonding produces higher gel viscosity at low polymer content. This research affirmed that rates of diffusion could be measured for residual water in polymer gels. It was shown, using DOSY, that the polymer gels themselves moved two orders of magnitude slower than the surrounding solvent (HOD). These rates were largely consistent with the bulk water rate of diffusion with slightly slower rates consistent with increased viscosity of the gel structures. Broadening on DOSY water peaks suggested that small populations of water were likely in chemical environments different from bulk water. This broadening phenomenon was larger for PEO than for either of the modified cellulose polymers and corroborated with DSC freezing studies, which implied a comparatively finer porous structure for PEO.

“Elastically” scattered neutrons from QENS measurements at BASIS indicated the mobility of hydrogen atoms of water molecules in a frozen state. The relationship between the slope of the neutron elastic intensity versus Temperature, and the Debye-Waller factor, were used to indicate relative differences in the modulus of frozen gels and frozen gels confined within cement paste. The modulus of neat HEMC gel was found to be higher than that for the other gel-forming polymers, consistent with rheological findings and the previously observed printing outcomes, which suggested that higher modulus gels produce better printing outcomes.

Dynamic data from QENS measurements at BASIS indicated that water diffuses slower within both neat gels and gel-cement than in bulk neat water, likely due to the presence of the polymer. This was somewhat inconsistent with DOSY findings that were unable to detect a significant difference between bulk water diffusion and diffusion in the presence of polymer.

## 4. Materials and Methods

### 4.1. Materials

Type I/II Portland cement was used and three different hydrogel-forming polymers were chosen: WALOCEL™ M-20678 hydroxyethyl methyl cellulose (HMEC), METHOCEL™ 240 hydroxypropyl methyl cellulose (HPMC), and DOW Chemical POLYOX™ WSR 301 polyethylene oxide (PEO). Gels made with the various polymers were referred to as: HEMC (Gel 1), HPMC (Gel 2), and PEO (Gel 3). Deionized water was used in all the experiments where water was required [34,35]. Deuterium oxide (D_2_O, 99.8 mass %) was used for all NMR DOSY and specific neutron scattering experiments. NMR diffusion experiments were done to measure both the mobility of the polymer and water.

To establish the water mobility, it was necessary to drop the concentration of water in the sample to within the detectable range of the instrument. Pure water would saturate the detector so D_2_O was used as a diluent. In addition, the D_2_O signal was used to adjust the magnetic field to be uniform through the sample; this common NMR practice is done by locking and shimming the instrument with D_2_O.

### 4.2. Gel Preparation Procedures

#### 4.2.1. Gels for Differential Scanning Calorimetry

To characterize the pore structure of hydrogels, 4.0 mass % hydrogels of the three selected polymers were prepared using procedures described in detail elsewhere [34,35]. In summary, gels were prepared by dispersing a requisite amount of the neat polymer into either cold or hot water and stirring vigorously until the solid was completely dispersed and a clear gel formed. Gels were then stored in sealed containers at 5 °C (refrigerated) until used. Gels were used within five days of preparation.

#### 4.2.2. Gels for NMR

Hydrogels containing 0.5 mass % of HEMC, 0.2 mass % of PEO, and 2.0 mass % of PEO were prepared using the procedure summarized above and detailed elsewhere [34,35]. Clean and dried NMR tubes were used for this experiment. Stainless steel syringe tips with a length of 15 mm and outer diameter of 3.5 mm were used to fill the NMR tubes with the prepared hydrogels. To reduce macroscopically entrapped air within the NMR tube, i.e., visible bubbles, a vacuum pump was used. Filled tubes were repeatedly placed under vacuum and then brought to atmospheric pressure until the majority of the visible air had been removed, typically requiring several cycles.

#### 4.2.3. Gels and Cement Pastes for Neutron Scattering

Gels used for neutron backscattering spectroscopy studies were prepared using deionized water, using the same procedures described previously [34,35]. Similarly, gel-cement pastes were prepared by hand mixing and shearing, using procedures also described in detail elsewhere [34,35].

### 4.3. Microstructural Analysis (XRD)

X-ray powder diffraction (XRD) was used for phase identification of the as-received polymers. The diffraction patterns were collected using Cu Kα radiation from a Rigaku Ultima IV diffractometer. The diffractometer was equipped with a D/tex Ultra-high-speed detector with fluorescence reduction. Diffraction patterns were collected from 20–80° 2θ in θ/2θ mode using a scan speed of 1°/min. The X-ray powder diffraction profiles for HEMC, HPMC, and PEO are shown in Figure 1. As expected, the PEO was highly ordered (crystalline), whereas the cellulose-derived materials were a combination of amorphous and crystalline matter characteristic of the parent cellulose [74,78,79,80].

### 4.4. Differential Scanning Calorimetry-Based Thermoporosimetry (DSC)

The melting behavior of water in the three hydrogels was investigated using thermoporosimetry. A DSC 2010 (Waters TA Instruments, New Castle, DE, USA) was used to investigate the pore structure and association between water and polymer in the hydrogels. Stainless steel pans with covers and O-rings (Perkin Elmer, Inc., Waltham, MA, USA) were used to prevent water evaporation. After carefully weighing the pans, covers, and O-rings, nominally 65 mg ± 5 mg of gel was placed inside a pan and the pan was hermetically sealed and again weighed. The pan was first cooled to −55 °C and then heated at a rate of 0.5 °C/min to 25 °C under a nitrogen purge gas flow of 40 cm^3^/min. DSC runs were done in triplicate to establish experimental error. The DSC heating graphs (melting curves) for HEMC, HPMC, PEO gels and deionized water are shown in Figure 2.

### 4.5. Diffusion-Ordered Nuclear Magnetic Resonance Spectroscopy (DOSY)

DOSY data was used to determine the self-diffusivity of residual partially deuterated water, i.e., deuterated water (HOD) in deuterium oxide (D_2_O) in neat hydrogels. DOSY experiments were conducted using a Advance ΙΙΙ 500 MHz NMR (Bruker, Billerica, MA, USA) with a liquid nitrogen cooled Prodigy Cryoprobe. This work used the standard Bruker pulse programs Stimulated Echo Sequence (stepg1s) pulse sequence and a one-dimensional (1D) longitudinal eddy current delay experiment using bipolar gradients (ledbpgp2s1d), along with the Bruker automation program for DOSY experiments. Self-diffusion coefficients were measured using a pulsed field gradient spin echo (PGSE) method [81,82,83,84,85,86]. DOSY experiments were performed at 25 °C. The DOSY spectra were acquired with the ledbpgp2s pulse program from Bruker TopSpin software. The gradient strength was logarithmically incremented in 32 steps from 5% up to 95% of the maximum gradient strength. All measurements were performed with a diffusion delay Δ = 100 ms to keep the relaxation contribution to the signal attenuation constant for all samples. The gradient pulse length δ was 5 ms in order to ensure full signal attenuation. The resulting NMR spectra were processed using Bruker Dynamics Center 2.5.6.b1, and DOSY maps were generated using Bruker Dynamics Center 2.5.6.b1 and MNova [87].

### 4.6. Quasi-Elastic Neutron Scattering

BASIS, a near-backscattering spectrometer at the Oak Ridge National Laboratory Spallation Neutron Source (ORNL/SNS) [88], was used in standard configuration, providing an energy resolution at the elastic line of ca. 3.7 µeV (full width at half maximum, FWHM) and accessible range of energy transfers of plus-minus 100 µeV. Besides measurements of the dynamic spectra (the neutron scattering intensity as a function of the energy and momentum transfer, I (Q, E)), BASIS was also used for measuring the temperature dependence of the energy-resolved “elastic” scattering intensity, as is customarily done for characterization of the phase state of the sample as a function of temperature [89]. Each sample was loaded into a flat-plate aluminum, indium-wire-sealed sample holder, 30 mm wide, 50 mm tall, and 0.5 mm thick. The temperature of the sample in the neutron beam was controlled with an accuracy of ± 0.25 K using a top-loading closed-cycle refrigerator. Loading of the sample holder was a somewhat tedious procedure which took roughly 30 min. An additional 15 min were used to place the loaded sample holder into the instrument. Samples were subsequently exposed to neutrons over a 2-h long dynamic measurement cycle to collect scattered intensities, I (Q, E). Thus, for each sample, the measurements of I (Q, E) at 300 K were completed in 2 h 45 min from the time that gel-cement samples were prepared or from the time that the neat gel samples were loaded. It should be noted that the procedure of preparation of neat gels and gel-cement- based pastes is described in detail elsewhere [35]. Following this measurement, the “elastic” intensity scans were collected, first on cooling to 10 K and then on warming at a rate of about 0.75 K/min. The timeline presented in Table 2 details the run cycle.

### 4.7. Atomistic Simulation

In support of the experimental observations, and to gain molecular-level insight into the structure and dynamics of hydrogel-forming polymers and their gels, atomistic molecular dynamic simulations (MDS) were performed for two cellulose-based polymers at various concentrations in water. A single polymer chain with 20 monomers was constructed using the Chain Builder module of Materials Studio and two such chains were packed into an orthogonal simulation box with periodic boundary conditions using the Amorphous Cell module. Water molecules were added to the initial box using the Grand Canonical Monte Carlo technique, in which water molecules are randomly inserted into the void spaces within the box. The number of water molecules was chosen to vary the concentration of polymers from 2 mass % to 20 mass %. The initial box size and the external pressure were chosen to be large enough to increase the probability of random insertion of water molecules. Once the required number of water molecules were added, the low-density system was subjected to energy minimization in a constant number, volume and temperature (NVT) ensemble, at 298.15 K, to relax the bonds, bond angles, and dihedrals of all constituents. This was then followed by a compression run using a constant number, pressure, and temperature (NPT) ensemble at 298.15 K and 1 atm to adjust the size of the simulation box corresponding to the correct density. The CVFF and SPC/E forcefields were used to model the polymers and water, respectively. The ensemble was then equilibrated at NVT for 50 ns with a timestep of 1 fs and energies were monitored to check for equilibration. The data for analyses were collected from additional production runs of about 10 ns. Whereas systems were constructed using Materials Studio, all simulations were carried out using the LAMMPS simulation package, including energy minimization, compression equilibration and production runs. This was done as a preference of the authors and could have been done a number of other ways.

## Figures and Tables

**Table 1:** Diffusion coefficients obtained from fit of the HWHM for *DQ*^2^ + offset.

Sample	Diffusivity, 10^−10^ m^2^/s	Elastic Signal Fraction
HEMC gel	13.9043 ± 0.2715	NA
HMPC gel	14.5941 ± 0.3757	NA
HMMC gel	16.3169 ± 0.3816	NA
PEO gel	14.0958 ± 0.2824	NA
cement	14.6989 ± 0.5890	1.729 × 10^−1^ ± 4.327 × 10^−3^
HEMC gel-cement	16.3777 ± 0.6809	1.562 × 10^−1^ ± 4.894 × 10^−3^
HMPC gel-cement	16.3929 ± 0.6986	1.523 × 10^−1^ ± 5.027 × 10^−3^
HMMC gel-cement	16.4233 ± 0.6736	1.698 × 10^−1^± 4.702 × 10^−3^
PEO gel-cement	16.3169 ± 0.6755	1.683 × 10^−1^ ± 5.321 × 10^−3^

**Table 2:** Timeline for the sample preparation and data collection for experiments at BASIS.

Procedure	Time
Preparation of gels and storage	Gel preparation procedure is described elsewhere [34,35].
Mixing gels with cement particles to make gel-cement paste	Between 5 to 10 min from time of first contact of gel and cement; the mixing procedure is described elsewhere [34,35].
Loading either neat gel or gel-cement sample into the holder before exposure to beam	Roughly 30 min to load and seal the holder.
Placing the sample in the instrument	Additional 15 min to load sample into the beam path (required the removal of previous sample).
Exposing sample to the beam	2 h for the dynamic runs, then an additional 12 to 24 h of exposure (depending upon neutron beam stability) to gather the freezing/melting elastic scan data.

## Data Availability

The data is available at: 10.6084/m9.figshare.21082264.
